# Transitional Neonatal Hypoglycemia and Adverse Neurodevelopment in Midchildhood

**DOI:** 10.1001/jamanetworkopen.2024.3683

**Published:** 2024-03-26

**Authors:** Marcia Roeper, Henrike Hoermann, Lisa M. Körner, Marvin Sobottka, Ertan Mayatepek, Sebastian Kummer, Thomas Meissner

**Affiliations:** 1 Department of General Pediatrics, Neonatology and Pediatric Cardiology, Medical Faculty, University Hospital Düsseldorf, Heinrich-Heine-University, Düsseldorf, Germany

## Abstract

**Importance:**

The circumstances under which neonatal hypoglycemia leads to brain damage remain unclear due to a lack of long-term data on the neurodevelopment of affected children. As a result, diagnostic strategies and treatment recommendations are inconsistent.

**Objective:**

To evaluate whether the occurrence of severe transitional neonatal hypoglycemia (defined as having at least 1 blood glucose measurement of 30 mg/dL or below) is associated with adverse neurodevelopment in midchildhood.

**Design, Setting, and Participants:**

This cohort study using neurodevelopmental testing of a retrospectively recruited cohort was conducted at a single-center tertiary hospital in Germany between March 2022 and February 2023. Children with neonatal blood glucose screening data were randomly selected from all births between 2010 and 2015. Frequency matching for sex, birth weight, gestational age, socioeconomic status, and primary risk factors for neonatal hypoglycemia was performed. Children with persistent hypoglycemia diseases or any risk factor for adverse neurodevelopment except hypoglycemia were excluded. Data were analyzed between February 2023 and March 2023.

**Exposure:**

At least 1 neonatal hypoglycemia measurement with blood glucose measuring 30 mg/dL or below vs all measured blood glucose levels above 30 mg/dL during postnatal blood glucose screening starting on the first day of life.

**Main Outcomes and Measures:**

Cognitive function measured by full-scale IQ test. Secondary outcomes included standardized scales of motor, visual, and executive functions, and child behavior, each measured at ages 7 to 11 years.

**Results:**

A total of 140 children (mean [SD] age 9.1 [1.3] years; 77 male [55.0%]) participated in the study. Children with severe neonatal hypoglycemia had a 4.8 points lower mean full-scale IQ than controls (107.0 [95% CI, 104.0-109.9] vs 111.8 [95% CI, 108.8-114.8]). They showed a 4.9-fold (95% CI, 1.5-15.5) increased odds of abnormal fine motor function and a 5.3-fold (95% CI, 2.1-13.3) increased odds of abnormal visual-motor integration. Significantly higher T scores for attention problems (58.2 [95% CI, 56.1-60.2] vs 54.6 [95% CI, 52.6-56.6]) and attention-deficit/hyperactivity disorder symptoms (58.2 [95% CI, 56.2-60.2] vs 54.7 [95% CI, 52.8-56.7]) were reported by parents.

**Conclusions and Relevance:**

Neonatal hypoglycemia with blood glucose levels of 30 mg/dL or below was associated with an increased risk for suboptimal neurodevelopmental outcomes in midchildhood. These findings imply that treatment strategies should aim to prevent episodes of hypoglycemia at these severely low levels.

## Key Points

**Question:**

Are episodes of severe transitional neonatal hypoglycemia associated with adverse neurodevelopment in midchildhood?

**Findings:**

In this cohort study of 140 children aged 7 to 11 years, a history of severe transitional neonatal hypoglycemia was associated with a 4.8 points lower full-scale IQ and significantly lower performance in visual-motor and fine motor functions.

**Meaning:**

These results suggest that severe transitional hypoglycemia is associated with adverse neurodevelopmental outcomes; treatment thresholds and goals for neonatal hypoglycemia should be high enough to prevent hypoglycemia at such levels from occurring.

## Teaser

This cohort study of German children aged 7 to 11 years examines the association of severe neonatal hypoglycemia with neurodevelopmental outcomes.

## Introduction

Hypoglycemia is the most common metabolic condition in neonates. The reported incidence is 15% in all neonates^1^ and 50% in babies born with risk factors, including infants of mothers with diabetes, those large or small for their gestational age, preterm births, or births with perinatal stress.^2^ The most common form is transitional neonatal hypoglycemia, which occurs during the metabolic transition from intrauterine to extrauterine life. Following the cessation of glucose supply through the umbilical cord after delivery, blood glucose levels drop to a nadir at 30 to 90 minutes postnatally, and then gradually rise to adult levels by the fourth day of life.^3,4,5,6^ In children with risk factors, hypoglycemia may be more severe and prolonged, or may occur later in the first days of life, eg, in case of lower glycogen reserves, increased glucose consumption, or increased insulin secretion.^7^ Therefore, neonates with risk factors usually undergo blood glucose screening to identify hypoglycemia early and treat it appropriately. However, guideline recommendations and treatment thresholds vary widely, particularly because it remains unclear what blood glucose levels are considered physiological in the first days of life and under what circumstances hypoglycemia can damage the neonatal brain.^8^

It is known that up to 50% of infants with hypoglycemia diseases, such as transient or congenital hyperinsulinism, may suffer brain injury.^9,10,11^ However, data on long-term neurodevelopmental outcomes beyond childhood for children with transitional neonatal hypoglycemia are limited. This is important because specific developmental consequences may not manifest until midchildhood or late childhood and may only be detected through developmental assessments. Therefore, the aim of this study was to evaluate whether transitional neonatal hypoglycemia with blood glucose measured at 30 mg/dL or lower (to convert glucose concentrations to millimoles per liter, multiply by 0.0555) is associated with adverse neurodevelopment in midchildhood compared with a control group.

## Methods

### Study Design

The ProBrain-D 7-11 Study is a matched cohort study that examined the neurodevelopmental outcomes of 140 children aged 7 to 11 years who were born in a tertiary hospital in Düsseldorf, Germany. Neurodevelopmental testing was prospectively conducted. The study was performed between March 2022 and February 2023.

Two groups were recruited based on retrospective data: 70 children (50%) with severe neonatal hypoglycemia, defined as a history of at least 1 recorded blood glucose level of 30 mg/dL or below (exposed group) and 70 children (50%) with all blood glucose levels above 30 mg/dL (unexposed group). Several guidelines suggest a threshold of 25 to 35 mg/dL (mean, 30 mg/dL) to initiate intense blood glucose treatment.^12,13,14,15,16,17,18,19,20,21^ However, none of these thresholds are currently supported by evidence. Additionally, studies using higher cutoffs, ie, between 36 and 47 mg/dL, have conflicting and inconclusive results regarding the neurodevelopmental consequences of hypoglycemia. Therefore, the cutoff of 30 mg/dL was intentionally selected for this study to provide evidence of neurological consequences at a lower threshold that usually triggers immediate action and intravenous glucose treatment.

To identify eligible patients, the database of all births in our hospital from 2010 to 2015 was first screened using *International Statistical Classification of Diseases and Related Health Problems, Tenth Revision (ICD-10)* codes (Figure). Of these, medical records were reviewed. The inclusion criterion was existing data on neonatal blood glucose screening, eg, due to suspected hypoglycemia or risk factors for hypoglycemia as defined by the hospital’s screening protocol (eAppendix in Supplement 1). Children with persistent hypoglycemia or a history of any risk factor or condition that could potentially cause adverse neurodevelopment other than hypoglycemia were excluded (Figure). All children were screened and treated for hypoglycemia according to the hospital’s protocol. Glucose concentrations were measured in whole blood by glucose oxidase-based methods, with glucose meters (Nova Biomedical) and blood gas analyzer (Radiometer).

**Figure.**
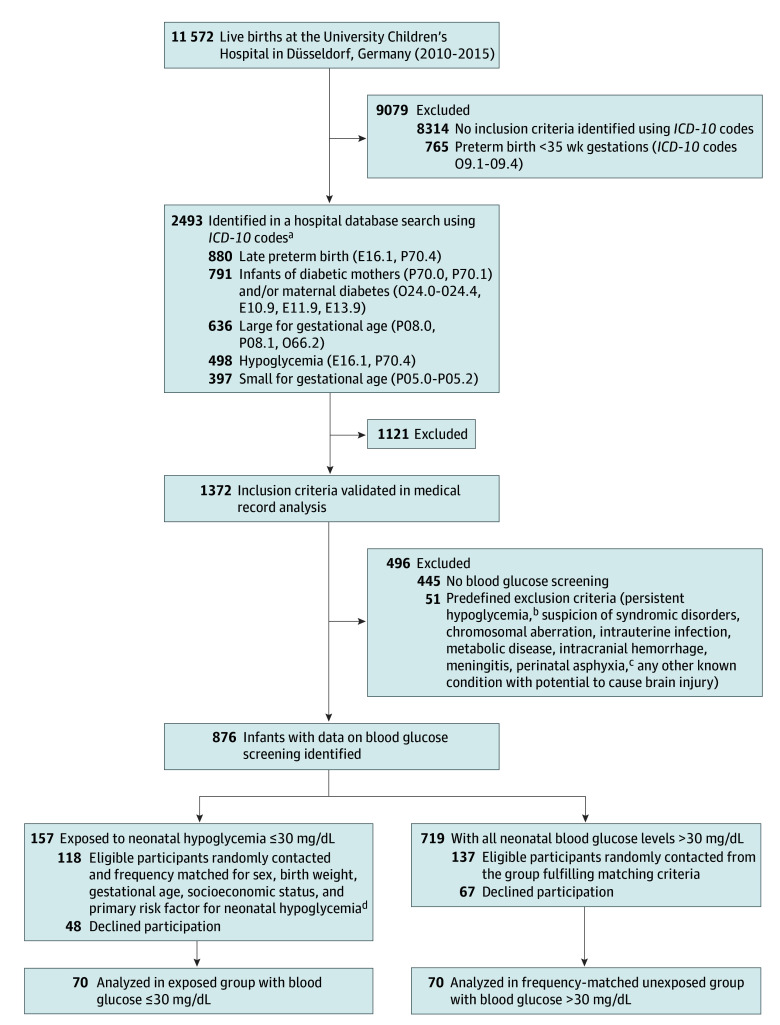
Flow Diagram *ICD-10* indicates *International Statistical Classification of Diseases and Related Health Problems, Tenth Revision*. To convert glucose to millimoles per liter, multiply by 0.0555. ^a^The total number of search results is displayed. Multiple search items were coded for some participants. ^b^Defined as requirement of any glucose-elevating medication beyond transitory carbohydrate supplementation, prolongation of hospital stay for blood glucose management, and/or diagnosis of a hypoglycemia disorder such as transient or persistent hyperinsulinism or hereditary metabolic disease. ^c^As defined by the guideline from Flemmer and colleagues^22^: perinatal stress plus at least 1 of the following parameters: pH < 7.0, base deficit greater than 16 mmol/L, Apgar score below 6 at 5 or 10 minutes. ^d^As defined by the hospital’s protocol for screening and treatment of neonatal hypoglycemia at the time of neonatal management in 2010-2015 (eAppendix in Supplement 1).

Recruitment was performed via telephone and email. Participants exposed to severe hypoglycemia were contacted in random order from the list of eligible individuals until the target number of 70 was recruited. In parallel, participants for the unexposed group were also contacted in random order from the eligible candidates, frequency matched to the exposure group for sex, birth weight, gestational age, socioeconomic status (SES) according to the SES Index,^23^ and primary risk factors for neonatal hypoglycemia. Parents were not informed of the group allocation or blood glucose cutoffs for group assignment at recruitment to avoid voluntary bias depending on exposure status. No incentives were offered for participation. Written informed consent was obtained from all parents along with childrens’ assent. The study was approved by the institutional review board of the Medical Faculty of the Heinrich-Heine-University Düsseldorf, Germany, and was performed following the Declaration of Helsinki.^24^ This report follows the Strengthening the Reporting of Observational Studies in Epidemiology (STROBE) reporting guideline for observational studies.

### Measures

Neurodevelopmental assessment was conducted by trained pediatricians or psychologists who were masked to the neonatal glycemic history of the children. Neurodevelopmental outcome tests were selected based on previously published studies to allow comparison of results. The predefined primary outcome parameter was the assessment of cognitive function (full-scale IQ) using Wechsler’s Intelligence Scale for Children–Fifth Edition (WISC-V) (standardized mean [SD], 100 [15]; below 85 points [ie, 15th percentile] was the cutoff for IQ below average).^25^ As prespecified secondary outcomes, motor function was assessed using Movement Assessment Battery for Children–Second Edition (MABC-2) (standardized mean [SD], 10 [3]; 6 points or below [10th percentile] was the cutoff for abnormal test result)^26^ and visual perception plus visual-motor function were evaluated using Developmental Test of Visual Perception–Third Edition/Adolescent and Adult (DTVP-3, DTVP-A) (standardized mean [SD], 100 [15]; below 85 points [15th percentile] was the cutoff for below average).^27^ Parallel to the developmental testing, ie, not knowing the test results or exposure status, parents completed the Behavior Rating Inventory of Executive Function (BRIEF) (standardized T score mean [SD], 50 [10]),^28^ the Child Behavior Checklist (CBCL) (standardized T score mean [SD], 50 [10]),^29^ and a questionnaire providing additional information regarding the child’s medical history, development and education, and the family’s SES.

Data were recorded in a digital database (Claris International Inc). The medical and birth history was obtained from the medical records of both the participants and their mothers. Furthermore, we reviewed data from the child’s last 2 school report cards and from the German child examination booklet, which contains all information on pediatric check-ups and developmental assessments. All data not required for recruitment or matching were collected after completion of developmental testing to avoid information and selection bias.

### Statistical Analysis

Sample size estimation was performed using G*Power version 3.1 (Heinrich-Heine-University).^30^ For the primary outcome parameter, we estimated a total sample size of at least 140 children (70 per group) to detect a true mean difference in IQ scores of 0.5 SD with a power of 0.9 at an α level of .05 using univariate analysis. Data were controlled for prespecified potential confounders by frequency matching the groups. Post hoc analysis of covariance was conducted to adjust the data for the remaining possible explanatory confounders that were assessed in the study but not already controlled for by matching, excluded by sensitivity analysis, or related to hypoglycemia. Unadjusted data are reported in eTable 1 in Supplement 1. Statistical analyses were performed using IBM SPSS Statistics version 28.0 (IBM Inc). Cohort characteristics were assessed using standard descriptive statistics (percentage, mean, median, 95% CI, SD, and IQR). Missing data were not imputed. Student *t* test was used to analyze parametric variables and a Mann-Whitney *U* test was used for nonparametric variables. Pearson χ^2^ test and Fisher exact test were used when appropriate to analyze associations between categorical variables. Mean differences (MD) or odds ratios (OR) are reported with a 95% CI for continuous or binary data, respectively. The effect size was evaluated using partial *η_p_^2^* (0.01 = small; 0.06 = medium; 0.14 = large effect).^31^ A 2-sided *P* < .05 was considered statistically significant. Adjustment for multiple comparisons was not applied because the study was only powered to test for significance of the primary end point at uncorrected *P* < .05. Regarding the subscores of the WISC, the main objective was to analyze which specific subscales are primarily affected by hypoglycemia and therefore contribute most to the observed effect in our primary end point. Similarly, secondary end points were assessed as exploratory.

## Results

A total number of 140 children participated in the study. Participation rate of those being contacted was similar in the exposed and unexposed group (70 of 118 [59.3%] vs 70 of 137 [51.1%]; *P* = .19). Mean (SD) age at time of assessment was 9.1 (1.3) years and 77 (55.0%) were male. Mean (SD) gestational age at birth was 37 weeks, 6 days (12 days) and mean (SD) birth weight was 3095 (948) g. In 107 neonates (76.4%), the first blood glucose measurement was performed within 2 hours of life, with a median (IQR) time of 69 (60-98) minutes in the exposed group and 79 (60-134) minutes in the unexposed group. The lowest blood glucose occurred in 86 (61.4%) neonates within the first 2 hours of life, in 39 (27.9%) between 2 and 12 hours of life, in 7 (7.1%) within 12 and 24 hours of life, and in 8 (5.7%) 24 hours or later after birth. In the exposed group, mean (SD) of lowest blood glucose was 22.3 (5.2) mg/dL and the lowest blood glucose level was measured at a median (IQR) age of 78 (60-120) minutes. In the unexposed group, mean (SD) of lowest blood glucose was 48.1 (12.1) mg/dL and the lowest blood glucose value was measured at a median (IQR) age of 123 (66-367) minutes. In the unexposed group, 37 children had no blood glucose level measurement below 45 mg/dL, and 33 had at least 1 blood glucose level between 31 and 45 mg/dL. No between-group differences were identified in terms of the predefined confounding factors, thus confirming adequacy of matching (Table 1).

**Table 1.** Characteristics of Study Participants

Child characteristics	Blood glucose ≤30 mg/dL, No. (%) (n = 70)	Blood glucose >30 mg/dL, No. (%) (n = 70)	*P* value
Sex			
Female	31 (44.3)	32 (45.7)	.87
Male	39 (55.7)	38 (54.3)	
Ethnicity			
European	59 (84.3)	65 (92.9)	.25
African	4 (5.7)	1 (1.4)	
Arabic	7 (10.0)	4 (5.7)	
Age at assessment, mean (SD), y	9.2 (1.4)	9.1 (1.2)	.66
Neonatal characteristics			
Gestational age, mean, wk + d (SD, d)	37 + 5 (12)	38 + 0 (12)	.36
Birth weight, mean (SD), g	3053 (924)	3136 (976)	.75
Birth weight, mean (SD), SDS	−0.22 (1.76)	−0.12 (1.79)	.79
Twins	21 (30)	18 (25.7)	.57
Cesarean delivery	48 (68.6)	41 (58.6)	.22
Apgar 1 min, median (IQR)	9 (8-9)	9 (8-9)	.62
Apgar 5 min, median (IQR)	10 (9-10)	10 (9-10)	.73
Apgar 10 min, median (IQR)	10 (10-10)	10 (10-10)	.77
Arterial cord blood pH, mean (SD)	7.27 (.08)	7.28 (.08)	.69
Base excess, mean (SD), mmol/L	−3.1 (3.4)	−3.4 (2.9)	.42
Childhood characteristics and development			
BMI at last check-up, mean (SD)	16.0 (1.8)	16.0 (1.9)	.95
No. of siblings, median (IQR)	1 (1-2)	1 (1-2)	.62
Having older siblings	30 (46.2)	28 (40.6)	.52
Breastfed	48 (68.5)	48 (68.5)	.79
Primary household language not German	7 (10)	4 (5.7)	.35
Attended school grade, median (IQR)	3 (2-4)	3 (2-4)	.71
Parent-reported history of developmental delay	21 (30)	15 (21.4)	.25
Parent-reported history of developmental therapy	28 (41.2)	31 (47.0)	.50
Parent-reported concentration and attention problems	28 (40)	14 (20)	.01
Risk factors for neonatal hypoglycemia			
No. of risk factors for neonatal hypoglycemia, median (IQR)	2 (1-2)	2 (1-3)	.47
Maternal diabetes in pregnancy	28 (40)	26 (37.1)	.73
SGA	24 (34.3)	23 (32.9)	.86
LGA	15 (21.4)	22 (31.4)	.18
Late preterm birth^a^	28 (40)	24 (34.3)	.48
Other/secondary risk factors for neonatal hypoglycemia	41 (58.6)	36 (51.4)	.40
**Blood glucose screening and treatment**			
No. of measurements, median (IQR)	12 (6-31)	4 (3-9)	<.001
Time of first measurement, median (IQR), min	69 (60-98)	79 (74)	.38
Lowest value, mean (SD), mg/dL	22.3 (5.2)	48.1 (12.1)	<.001
Age at lowest value, median (IQR), min	78 (60-120)	123 (66-367)	.001
Duration of screening, median (IQR), h	42.9 (12.5-101.3)	13.1 (9.4-38.3)	<.001
Mean (SD) measure by time interval, mg/dL			
<12 h	53.4 (9.9)	62.6 (10.1)	<.001
12 - 24 h	63.7 (11.8)	65.0 (10.4)	.64
24-48 h	63.6 (7.9)	68.3 (13.1)	.10
>48 h	67.3 (7.5)	70.2 (12.3)	.40
All measurements	59.1 (7.7)	64.9 (9.4)	<.001
No. of measurements by blood glucose level, median (IQR)			
≤30 mg/dL	1 (1-1)	0	<.001
31-45 mg/dL	1 (0-3)	0 (0-2)	<.001
46-54 mg/dL	2 (1-4)	1 (0-2)	.002
Children with recurrent severe hypoglycemia^b^	21 (30)	0	<.001
IV glucose treatment	33 (47.1)	11 (15.7)	<.001
Duration of IV glucose treatment, mean (SD), d	4.0 (1.8)	3.0 (1.9)	.13
Maximum GIR, median (IQR), mg/kg/min	4.5 (3.1-5.3)	3.1 (1.9-5.0)	.10
Treatment in neonatal unit for reasons other than hypoglycemia^c^	16 (22.9)	21 (30.0)	.34
Treatment in neonatal unit due to hypoglycemia	32 (45.7)	8 (11.4)	<.001
Maternal and parental characteristics			
Maternal age at birth of child, mean (SD), y	33.5 (4.5)	32.8 (4.8)	.39
Maternal BMI before pregnancy (n = 130), mean (SD)	25.1 (5.0)	25.9 (6.3)	.96
Maternal BMI before birth, mean (SD)	30.0 (5.0)	29.9 (5.8)	.70
Gravity, median (IQR)	2 (1-3)	2 (1-2)	.85
Parity, median (IQR)	1 (1-2)	1 (1-2)	.17
Smoking in pregnancy	6 (8.6)	2 (2.9)	.28
Alcohol use or substance abuse in pregnancy	0	0	NA
Socioeconomic status index score, median (IQR)^d^	17.8 (15.7-19.1)	18 (16.2-20.0)	.13
Parental highest educational level college or university	45 (64.3)	45 (64.3)	.72

Abbreviations: BMI, body mass index (calculated as weight in kilograms divided by height in meters squared); GIR, glucose infusion rate; IV, intravenous; LGA, large for gestational age; NA, not applicable; SDS, standard deviation score; SGA, small for gestational age.

SI conversion factor: To convert blood glucose concentration to millimoles per liter, multiply values by 0.0555.

^a^ Including births from 35 weeks, 0 days to 36 weeks, 6 days.

^b^ Recurrent hypoglycemia was defined as ≥2 episodes; severe hypoglycemia defined as blood glucose measurements of 30 mg/dL or below.

^c^ Sensitivity analysis for the item did not alter the primary conclusion.

^d^ Score range: 3.2 to 21.0. Higher scores indicate higher socioeconomic status.^23^

### Neurodevelopmental Outcomes

Children in the exposed group had a 4.8 points lower WISC-V mean full-scale IQ than the unexposed group (107.0 [95% CI, 104.0-109.9] vs 111.8 [95% CI, 108.8-114.8]). Significantly lower mean WISC-V subtest scores were observed for verbal comprehension (108.9 [95% CI, 105.8-111.9] vs 113.8 [95% CI, 110.7-116.8]) and processing speed (100.5 [95% CI, 97.1-103.9] vs 106.3 [95% CI, 102.9-109.7]) (Table 2). In the MABC-2, exposed children had significantly worse scores for total motor function (9.3 [95% CI, 8.7-10.0] vs 10.3 [95% CI, 9.7-11.0]), mainly due to a significantly poorer performance on the test of fine motor function (9.3 [95% CI, 8.5-10.1] vs 10.8 [95% CI, 10.0-11.5]), and significantly more children had abnormal test results below the 10th percentile (16 [22.9%] vs 4 [5.7%]; OR, 4.9 [95% CI, 1.5 to 15.5]) (Table 3). There were no differences in balance, but both groups had difficulty with the gross motor tests (Table 2). On the DTVP, exposed children had significantly lower visual-motor integration test scores (93.0 [95% CI, 89.4-96.5] vs 103.3 [95% CI, 99.7-106.8]), and higher odds of an abnormal test score below the 15th percentile in this domain (26 [37.1%] vs 7 [10.0%]; OR, 5.3 [95% CI, 2.1 to 13.3]). The score for general visual perception was also significantly lower in the exposed group (94.6 [95% CI, 91.2-98.0] vs 101.2 [95% CI, 97.8-104.6]), but this was mainly driven by the lower score for visual-motor function. Parental ratings of executive function using the BRIEF questionnaire revealed significant differences between the groups only on the working memory subscale (adjusted mean T score, 51.9 [95% CI, 49.4-54.3] vs 48.2 [95% CI, 45.8-50.6]). On the CBCL, parent reports resulted in significantly higher T scores for the attention problems scale (58.2 [95% CI, 56.1-60.2] vs 54.6 [95% CI, 52.6-56.6]) and, correspondingly, for the attention-deficit/hyperactivity disorder (ADHD) scale (58.2 [95% CI, 56.2-60.2] vs 54.7 [95% CI, 52.8-56.7]). Recurrent blood glucose measurements of 30 mg/dL or below (ie, 2 or more episodes; mean [SD], 2.9 [1.2] episodes) were not associated with significantly worse outcomes than a single blood glucose value 30 mg/dL or below (eTable 2 in Supplement 1).

**Table 2.** Association of Neonatal Hypoglycemia and Neurodevelopmental Outcomes in Midchildhood^a^

Neurodevelopmental test	Adjusted mean (95% CI)		Adjusted mean difference (95% CI)	Adjusted *P* value	η*_p_*^2 ^^b^
	Blood glucose ≤30 mg/dL	Blood glucose >30 mg/dL			
WISC-V IQ (index score) (n = 137)^c^					
Total score	107.0 (104.0-109.9)	111.8 (108.8-114.8)	−4.8 (−9.0 to −0.6)	.03	.04
Verbal comprehension	108.9 (105.8-111.9)	113.8 (110.7-116.8)	−4.9 (−9.2 to −0.6)	.03	.04
Visual spatial	103.5 (100.1-106.8)	106.9 (103.6-110.3)	−3.5 (−8.2 to 1.3)	.15	NR
Fluid reasoning	106.2 (102.7-109.7)	107.9 (103.9-110.7)	−1.7 (−6.7 to 3.2)	.49	NR
Working memory	105.8 (102.4-109.2)	107.3 (103.8-111.2)	−1.5 (−6.3 to 3.4)	.55	NR
Processing speed	100.5 (97.1-103.9)	106.3 (102.9-109.7)	−5.8 (−10.6 to −0.9)	.02	.04
MABC-2 motor function (standard score) (n = 137)^d^					
Total motor function	9.3 (8.7-10.0)	10.3 (9.7-11.0)	−1.0 (−1.9 to −0.1)	.03	.04
Fine motor function	9.3 (8.5-10.1)	10.8 (10.0-11.5)	−1.5 (−2.5 to −0.4)	.006	.06
Gross motor function	8.4 (7.7-9.1)	8.8 (8.1-9.5)	−0.4 (−1.4 to 0.5)	.38	NR
Balance	10.4 (9.7-11.0)	11.0 (10.3-11.7)	−0.7 (−1.6 to 0.3)	.17	NR
DTVP visual perception (index score) (n = 137)^e^					
General visual perception	94.6 (91.2-98.0)	101.2 (97.8-104.6)	−6.6 (−11.4 to −1.8)	.008	.05
Motor-reduced visual perception	96.6 (93.0-100.3)	99.4 (95.7-103.0)	−2.7 (−7.9 to 2.5)	.30	NR
Visual-motor integration	93.0 (89.4-96.5)	103.3 (99.7-106.8)	−10.3 (−15.3 to −5.3)	<.001	.11
BRIEF (parental rated T scores) (n = 135)^f^					
Inhibit	50.4 (48.2-52.6)	48.8 (46.6-51.0)	1.6 (−1.5 to 4.8)	.30	NR
Shift	47.8 (45.6-49.9)	48.8 (46.6-50.9)	−1.0 (−4.0 to 2.1)	.52	NR
Emotional control	47.5 (45.4-49.6)	47.3 (45.1-49.3)	0.2 (−2.8 to 3.2)	.88	NR
Initiate	52.7 (50.1-55.4)	51.2 (48.6-53.8)	1.5 (−2.2 to 5.2)	.42	NR
Working memory	51.9 (49.4-54.3)	48.2 (45.8-50.6)	3.7 (0.2 to 7.1)	.04	.03
Plan/organize	50.9 (48.7-53.1)	47.9 (45.8-50.1)	3.0 (−0.09 to 6.1)	.06	NR
Organization of materials	50.8 (48.3-53.4)	49.6 (47.0-52.1)	1.3 (−2.3 to 4.9)	.49	NR
Monitor	49.8 (47.6-52.0)	49.5 (47.3-51.7)	0.3 (−2.8 to 3.4)	.84	NR
Behavioral regulation index	48.6 (46.4-50.8)	48.1 (45.8-50.3)	0.5 (−2.6 to 3.7)	.73	NR
Metacognition index	51.4 (49.1-53.7)	49.1 (46.7-51.4)	2.3 (−1.0 to 5.6)	.17	NR
Global executive composite	50.4 (48.1-52.6)	48.4 (46.2-50.7)	1.6 (−1.2 to 5.2)	.23	NR
CBCL (parental rated T scores) (n = 133)^g^					
Syndrome scales					
Anxious/depressed	56.9 (55.2-58.6)	56.4 (54.7-58.1)	0.5 (−1.9 to 2.9)	.67	NR
Depressed	55.4 (53.9-56.8)	53.1 (51.7-54.5)	2.3 (0.2 to 4.3)	.03	.04
Somatic complaints	55.2 (53.5-56.9)	55.6 (53.9-57.2)	−0.4 (−2.7 to 2.0)	.76	NR
Social problems	56.1 (54.5-57.7)	54.6 (53.1-56.2)	1.5 (−0.8 to 3.7)	.20	NR
Thought problems	57.6 (56.0-59.3)	56.5 (54.9-58.1)	1.1 (−1.2 to 3.5)	.33	NR
Attention problems	58.2 (56.1-60.2)	54.6 (52.6-56.6)	3.6 (0.7 to 6.4)	.02	.05
Rule-breaking behavior	54.8 (53.4-56.2)	55.1 (53.8-56.5)	−0.3 (−2.3 to 1.6)	.74	NR
Aggressive behavior	55.3 (53.5-57.0)	54.1 (52.4-55.8)	1.1 (−1.3 to 3.6)	.37	NR
Internalizing	54.3 (52.0-56.6)	53.4 (51.1-55.6)	0.9 (−2.3 to 4.2)	.56	NR
Externalizing	51.4 (48.9-53.9)	50.9 (48.5-53.4)	0.5 (−3.1 to 4.0)	.80	NR
Total problems	54.3 (51.8-56.7)	51.1 (49.7-54.5)	2.1 (−1.3 to 5.6)	.23	NR
*DSM*-oriented scales					
Affective problems	56.0 (54.4-57.5)	53.8 (52.3-55.4)	2.1 (−0.1 to 4.3)	.06	NR
Anxiety problems	57.3 (55.6-59.0)	57.0 (55.3-58.7)	0.3 (−2.1 to 2.7)	.82	NR
Somatic problems	54.9 (53.2-56.7)	54.7 (52.9-56.4)	0.3 (−2.2 to 2.8)	.83	NR
ADHD	58.2 (56.2-60.2)	54.7 (52.8-56.7)	3.5 (0.7 to 6.3)	.02	.05
Oppositional defiant problems	54.9 (53.2-56.6)	54.6 (53.0-56.2)	0.3 (−2.0 to 2.6)	.80	NR
Conduct problems	55.2 (53.7-56.8)	54.7 (53.2-56.3)	0.5 (−1.7 to 2.7)	.66	NR

Abbreviations: ADHD, attention deficit hyperactivity disorder; BRIEF, Behavior Rating Inventory of Executive Function; CBCL, Child Behavior Checklist; *DSM*, *Diagnostic and Statistical Manual of Mental Disorders*; DTVP, Developmental Test of Visual Perception; MABC-2, Movement Assessment Battery for Children–Second Edition; NR, not reported; WISC-V, Wechsler’s Intelligence Scale for Children–Fifth Edition.

SI conversion factor: To convert blood glucose concentration to millimoles per liter, multiply values by 0.0555.

^a^ Results from analyses of covariance adjusting for potential confounding by parental highest educational level, maternal smoking in pregnancy and breastfeeding are reported. Missing data were not imputed: 3 missing data on breastfeeding, 3 on BRIEF score, and 4 on CBCL score.

^b^ Partial eta-squared (η_p_^2^) only reported for significant values (.01 = small; .06 = medium; .14 = large effect).

^c^ WISC-V standardized mean (SD), 100 (15). Higher scores indicate higher IQ.

^d^ MABC-2 standardized mean (SD), 10 (3). Higher scores indicate higher function.

^e^ DTVP–Third Edition/Adolescent and Adult standardized mean (SD), 100 (15). Higher scores indicate higher function.

^f^ BRIEF standardized T score mean (SD), 50 (10). Higher scores indicate greater problems.

^g^ CBCL standardized T score mean (SD), 50 (10). Higher scores indicate greater problems.

**Table 3.** Association Between Neonatal Hypoglycemia and Underachievement on Neurodevelopmental Tests in Midchildhood

Characteristic	Participants, No. (%)		Odds ratio (95% CI)	*P* value
	Blood glucose ≤30 mg/dL	Blood glucose >30 mg/dL		
WISC-V IQ <15th percentile^a^				
Total score	4 (5.7)	2 (2.9)	2.1 (0.4-11.6)	.68
Verbal comprehension	4 (5.7)	1 (1.4)	4.2 (0.5-38.4)	.37
Visual spatial	7 (10)	3 (4.3)	2.4 (0.6-10.0)	.19
Fluid reasoning	7 (10)	3 (4.3)	2.4 (0.6-10.0)	.19
Working memory	4 (5.7)	5 (7.1)	0.8 (0.2-3.2)	>.99
Processing speed	10 (14.3)	2 (2.9)	5.7 (1.2-26.9)	.02
MABC-2 motor function <10th percentile^b^				
Total motor function	12 (17.1)	4 (5.7)	3.4 (1.04-11.2)	.03
Fine motor function	16 (22.9)	4 (5.7)	4.9 (1.5-15.5)	.004
Gross motor function	18 (25.7)	16 (22.9)	1.2 (0.5-2.5)	.69
Balance	8 (11.4)	2 (2.9)	4.4 (0.9-21.5)	.05
DTVP visual perception <15th percentile^c^				
General visual perception	17 (24.6)	11 (15.7)	1.8 (0.8-4.1)	.19
Motor-reduced visual perception	14 (20.3)	19 (27.1)	0.7 (0.3-1.5)	.34
Visual-motor integration	26 (37.1)	7 (10)	5.3 (2.1-13.3)	<.001

Abbreviations: DTVP, Developmental Test of Visual Perception; MABC-2, Movement Assessment Battery for Children–Second Edition; WISC-V, Wechsler’s Intelligence Scale for Children–Fifth Edition.

SI conversion factor: To convert blood glucose concentration to millimoles per liter, multiply values by 0.0555.

^a^ WISC-V cutoff for below average score was 15th percentile (below 85 index point).

^b^ MABC-2 cutoff for abnormal test result was below 10th percentile (below 6 standard points or under).

^c^ DTVP–Third Edition/Adolescent and Adult cutoff for result below average was 15th percentile (below 85 index point).

## Discussion

Here, we compare the standardized neurodevelopmental assessment of 140 children aged 7 to 11 years, with and without a history of severe transitional neonatal hypoglycemia. Children exposed to severe hypoglycemia had a 4.8 points lower full-scale IQ on the WISC-V when compared with a matched group of unexposed children. Furthermore, exposed children had significantly lower scores for visual-motor function, general visual perception, fine motor function, as well as total motor function. Although within the higher normal range, parent-reported scores for ADHD symptoms were significantly higher in the exposed group.

Few studies have examined the neurodevelopmental outcomes of school-aged children after neonatal hypoglycemia using standardized measures. One retrospective study^32^ found no significant difference in WISC-IV full-scale IQ at ages 6 to 9 years between 71 children after severe neonatal hypoglycemia and 32 control siblings. However, the study was not powered to detect differences of less than 8.4 IQ points, and blood glucose values were not reported for the control group because the only inclusion criterion was “no hospital report of neonatal hypoglycemia.” Consistent with our findings, there were lower scores for fine motor function on the MABC-2 in the hypoglycemia group, and no significant differences for gross motor function or balance.

Two studies have evaluated academic performance in midchildhood after neonatal hypoglycemia.^33,36^ However, academic performance may be confounded by various factors, such as the quality and conditions of education. After controlling for confounders, test scores on a fourth-grade achievement test in 1395 ten-year-old children revealed an association of neonatal hypoglycemia with glucose levels between 40 mg/dL and 45 mg/dL with decreased proficiency in mathematics and literacy, respectively.^33^ However, glycemic and developmental data were retrospective, many childhood characteristics were unknown (eg, primary household language, disabilities), only the first 2 blood glucose values were evaluated, and the lowest blood glucose value or treatment strategies were not reported.

The CHYLD study (Children With Hypoglycemia and Their Later Development) followed infants at risk for hypoglycemia prospectively with repeated neurodevelopmental assessment until age 10 years.^34,35,36^ All children underwent blood glucose screening after birth and were treated to maintain blood glucose levels above 47 mg/dL. There were no differences in neurodevelopmental outcomes between children with neonatal hypoglycemia who had glucose levels below 47 mg/dL and euglycemic infants at 2 years of age.^35^ However, consistent with our findings, neonatal hypoglycemia at that glucose level correlated with an increased risk of poor visual-motor function and poor executive function at 4.5 years, especially in those with recurrent hypoglycemia or severe episodes with glucose measured below 36 mg/dL.^34^ At 9 and 10 years of age, educational achievements and other neurodevelopmental domains were not significantly different between the groups. In contrast to our cohort, both groups showed low overall performance, academic achievements, fine- and visual-motor functions, emotional behavior regulation, and executive functions.^36^ It was hypothesized that the underlying risk factor for hypoglycemia may have a greater impact on neurodevelopmental trajectories than hypoglycemia itself.

A 2020 prospective randomized clinical trial^37^ compared a lower treatment threshold (36 mg/dL) with a traditional threshold (47 mg/dL) in asymptomatic at-risk neonates with initial blood glucose values above 35 mg/dL. Neurodevelopmental assessment at 18 months of age yielded no difference between groups. It is noteworthy that neonates with initial severe hypoglycemia below 35 mg/dL—the very patients shown to have inferior outcomes in our analysis—were excluded from the study. Furthermore, specific developmental difficulties may not present until later in life.^38^

In our cohort, 61.4% of neonates had their lowest blood glucose level within the first 2 hours of life. This suggests that severe hypoglycemia may result in adverse outcomes even at the time of physiological nadir. Preventive measures, such as early breastfeeding and prevention of hypothermia, should therefore be considered.

Our study supports that visual-motor functions are particularly affected by neonatal hypoglycemia. These are largely represented in the primary visual cortex in the occipital and parietal lobes, where structural damage following neonatal hypoglycemia has been described frequently, although the underlying mechanism for this spatial association remains unidentified.^39^

Notably, although we show that neonatal blood glucose levels of 30 mg/dL or below are associated with adverse effects on neurodevelopment, this does not imply that values above 30 mg/dL are completely safe, or that the threshold for potential brain damage is exactly 30 mg/dL. Brain injury depends on several factors, including the duration of low blood glucose, the concentration of other energy-providing metabolites such as ketone bodies and lactate, and the energy reserves in the brain. Therefore, multicenter studies are necessary to progressively evaluate the results of different intervention thresholds.

### Strengths and Limitations

Strengths of this study include the matched cohort design to adjust for potential confounders, and the prospective neurodevelopmental assessment of cognition, motor function, visual function, executive function, and child behavior using standardized measures by investigators masked to neonatal glycemia, powered to allow the detection of small, but still clinically significant, between-group differences. Treatment and screening for hypoglycemia was similar for all children because a single-center protocol was used.

This study has some limitations. First, because neonatal glycemia data are retrospective, granularity and accuracy may be compromised compared with a study design capturing neonatal data prospectively. Second, despite matching and adjustment for confounders of the primary outcome, we cannot rule out residual confounders that were not accounted for in this study. Third, the study was only powered to test for significance of the primary end point at uncorrected *P* < .05. Therefore, all secondary exploratory end points need to be interpreted cautiously and require further evaluation in confirmatory studies.

Fourth, high SES, above average full-scale IQ, and a high parental educational level are overrepresented in both groups, which limits generalizability of the results. However, after careful group matching and data adjustment, this aspect should not bias the between-group differences in terms of neurodevelopmental outcomes. Fifth, the low overall performance on the gross motor function test could be attributed to the extended cancellation of physical education classes and limited social and interactive play throughout the COVID-19 pandemic.

## Conclusions

Our data suggest that the group of neonates with severe hypoglycemia measured at 30 mg/dL or below was associated with an increased blood glucose–related risk of suboptimal neurodevelopmental outcomes. Therefore, treatment strategies should aim to prevent episodes of neonatal hypoglycemia at these levels until further data are available on intervention thresholds and neurodevelopmental outcomes in school-aged children from prospective and randomized multicenter trials.

## Notes

Supplement 1. **eAppendix.** Standard Operating Procedure for Neonatal Hypoglycemia at the University Children’s Hospital Düsseldorf, Germany, 2010-2015 **eTable 1.** Association of Neonatal Hypoglycemia and Neurodevelopmental Outcomes in Midchildhood (Unadjusted Data) **eTable 2.** Neurodevelopmental Outcomes After Singular or Recurrent Hypoglycemia ≤30 mg/dL

Supplement 2.
Data Sharing Statement

## References

[1] Hay WW Jr, Raju TN, Higgins RD, Kalhan SC, Devaskar SU. Knowledge gaps and research needs for understanding and treating neonatal hypoglycemia: workshop report from Eunice Kennedy Shriver National Institute of Child Health and Human Development. J Pediatr. 2009;155(5):612-617. doi:10.1016/j.jpeds.2009.06.044 19840614 PMC3857033

[2] Harris DL, Weston PJ, Harding JE. Incidence of neonatal hypoglycemia in babies identified as at risk. J Pediatr. 2012;161(5):787-791. doi:10.1016/j.jpeds.2012.05.022 22727868

[3] Harris DL, Weston PJ, Gamble GD, Harding JE. Glucose profiles in healthy term infants in the first 5 days: the Glucose in Well Babies (GLOW) Study. J Pediatr. 2020;223:34-41.e4. doi:10.1016/j.jpeds.2020.02.079 32381469

[4] Cornblath M, Reisner SH. Blood glucose in the neonate and its clinical significance. N Engl J Med. 1965;273(7):378-381. doi:10.1056/NEJM196508122730707 21417085

[5] Srinivasan G, Pildes RS, Cattamanchi G, Voora S, Lilien LD. Plasma glucose values in normal neonates: a new look. J Pediatr. 1986;109(1):114-117. doi:10.1016/S0022-3476(86)80588-1 3723230

[6] Hawdon JM, Ward Platt MP, Aynsley-Green A. Patterns of metabolic adaptation for preterm and term infants in the first neonatal week. Arch Dis Child. 1992;67(4 Spec No):357-365. doi:10.1136/adc.67.4_Spec_No.357 1586171 PMC1590511

[7] Vain NE, Chiarelli F. Neonatal hypoglycaemia: a never-ending story? Neonatology. 2021;118(5):522-529. doi:10.1159/000514711 33752207

[8] Roeper M, Hoermann H, Kummer S, Meissner T. Neonatal hypoglycemia: lack of evidence for a safe management. Front Endocrinol (Lausanne). 2023;14:1179102. doi:10.3389/fendo.2023.1179102 37361517 PMC10285477

[9] Meissner T, Wendel U, Burgard P, Schaetzle S, Mayatepek E. Long-term follow-up of 114 patients with congenital hyperinsulinism. Eur J Endocrinol. 2003;149(1):43-51. doi:10.1530/eje.0.1490043 12824865

[10] Helleskov A, Melikyan M, Globa E, . Both low blood glucose and insufficient treatment confer risk of neurodevelopmental impairment in congenital hyperinsulinism: a multinational cohort study. Front Endocrinol (Lausanne). 2017;8:156. doi:10.3389/fendo.2017.00156 28740482 PMC5502348

[11] Roeper M, Salimi Dafsari R, Hoermann H, Mayatepek E, Kummer S, Meissner T. Risk factors for adverse neurodevelopment in transient or persistent congenital hyperinsulinism. Front Endocrinol (Lausanne). 2020;11:580642. doi:10.3389/fendo.2020.580642 33424766 PMC7793856

[12] Queensland Government. Clinical Guidelines—Hypoglycaemia-newborn. Guideline No. MN23.8-V13-R28. Queensland Health. Last updated November 2023. Accessed January 8, 2024. www.health.qld.gov.au/__data/assets/pdf_file/0043/881899/g-hypogly.pdf

[13] Waitemata District Health Board. Neonatal Hypoglycaemia Guideline. July 2021. Accessed January 8, 2024. https://www.healthpoint.co.nz/download,3daa10cd-a318-48ce-a396-dfafe3dd8c79.do

[14] Government of Western Australia, Child and Adolescent Health Service. Hypoglycaemia Guideline. Reviewed December 2023. Accessed January 8, 2024. https://pch.health.wa.gov.au/For-health-professionals/Emergency-Department-Guidelines/Hypoglycaemia

[15] Varghese J, McPhee A, Morris S, Stewart N, . South Australian Perinatal Practice Guideline. Neonatal Hypoglycaemia. Department for Health and Wellbeing, Government of South Australia. Reviewed September 2020. Accessed January 8, 2024. https://www.sahealth.sa.gov.au/wps/wcm/connect/8b64d0004ee49f7584608dd150ce4f37/Neonatal+Hypoglycaemia_PPG_v5_0.pdf

[16] Wackernagel D, Gustafsson A, Edstedt Bonamy AK, . Swedish national guideline for prevention and treatment of neonatal hypoglycaemia in newborn infants with gestational age ≥35 weeks. Acta Paediatr. 2020;109(1):31-44. doi:10.1111/apa.14955 31350926

[17] Narvey MR, Marks SD. The screening and management of newborns at risk for low blood glucose. Paediatr Child Health. 2019;24(8):536-554. doi:10.1093/pch/pxz134 31844395 PMC6901164

[18] Aliefendioglu D, Coban A, Hatipoglu N, . Management of hypoglycemia in newborn: Turkish Neonatal and Pediatric Endocrinology and Diabetes Societies consensus report. Turk Pediatri Ars. 2018;53(Suppl 1):S224-S233. doi:10.5152/TurkPediatriArs.2018.01820 31236035 PMC6568301

[19] Adamkin DH; Committee on Fetus and Newborn. Postnatal glucose homeostasis in late-preterm and term infants. Pediatrics. 2011;127(3):575-579. doi:10.1542/peds.2010-3851 21357346

[20] Rasmussen AH, Wehberg S, Fenger-Groen J, Christesen HT. Retrospective evaluation of a national guideline to prevent neonatal hypoglycemia. Pediatr Neonatol. 2017;58(5):398-405. doi:10.1016/j.pedneo.2016.12.002 28237510

[21] Segerer H, Bührer C, Kapellen T, . Betreuung von Neugeborenen diabetischer Mütter. [Care of Infants of Diabetic Mothers]. Z Geburtshilfe Neonatol. 2018;222(03):107-114. doi:10.1055/a-0628-0873 29920628

[22] Flemmer AW, Maier RF, Hummler H. Behandlung der neonatalen Asphyxie unter besonderer Berücksichtigung der therapeutischen Hypothermie [Treatment of neonatal asphyxia with a special focus on therapeutic hypothermia]. Klin Padiatr. 2014;226(1):29-37. doi:10.1055/s-0033-1361104 24435792

[23] Lampert T, Hoebel J, Kuntz B, Müters S, Kroll LE. Socioeconomic status and subjective social status measurement in KiGGS Wave 2. J Health Monit. 2018;3(1):108-125. doi:10.17886/RKI-GBE-2018-033 35586179 PMC8848848

[24] World Medical Association. World Medical Association Declaration of Helsinki: ethical principles for medical research involving human subjects. JAMA. 2013;310(20):2191-2194. doi:10.1001/jama.2013.281053 24141714

[25] Petermann F. Wechsler Intelligence Scale for Children—Fifth Edition (WISC-V). Pearson; 2017.

[26] Petermann F. Movement Assessment Battery for Children-2. Pearson; 2015. 10.1016/j.ridd.2010.11.016 21146955

[27] Büttner G. Frostigs Entwicklungstest der visuellen Wahrnehmung–3 (FEW-3). Hogrefe; 2021.

[28] Drechsler R, Steinhausen HC. Verhaltensinventar zur Beurteilung exekutiver Funktionen (BRIEF). Hogrefe; 2013.

[29] Döpfner M, Plück J, Kinnen C. Deutsche Schulalter-Formen der Child Behavior Checklist. Hogrefe; 2014.

[30] Faul F, Erdfelder E, Lang AG, Buchner A. G*Power 3: a flexible statistical power analysis program for the social, behavioral, and biomedical sciences. Behav Res Methods. 2007;39(2):175-191. doi:10.3758/BF03193146 17695343

[31] Cohen J. Statistical power analysis for the behavioural sciences. Erlbaum; 1988.

[32] Rasmussen AH, Wehberg S, Pørtner F, Larsen AM, Filipsen K, Christesen HT. Neurodevelopmental outcomes after moderate to severe neonatal hypoglycemia. Eur J Pediatr. 2020;179(12):1981-1991. doi:10.1007/s00431-020-03729-x 32666280 PMC7666672

[33] Kaiser JR, Bai S, Gibson N, . Association between transient newborn hypoglycemia and fourth-grade achievement test proficiency: a population-based study. JAMA Pediatr. 2015;169(10):913-921. doi:10.1001/jamapediatrics.2015.1631 26301959

[34] McKinlay CJD, Alsweiler JM, Anstice NS, ; Children With Hypoglycemia and Their Later Development (CHYLD) Study Team. Association of neonatal glycemia with neurodevelopmental outcomes at 4.5 years. JAMA Pediatr. 2017;171(10):972-983. doi:10.1001/jamapediatrics.2017.1579 28783802 PMC5710616

[35] Harris DL, Alsweiler JM, Ansell JM, . Outcome at 2 years after dextrose gel treatment for neonatal hypoglycemia: follow-up of a randomized trial. J Pediatr. 2016;170:54-59.E2. doi:10.1016/j.jpeds.2015.10.066 26613985 PMC4769950

[36] Shah R, Dai DWT, Alsweiler JM, ; Children With Hypoglycaemia and Their Later Development (CHYLD) Study Team. Association of neonatal hypoglycemia with academic performance in mid-childhood. JAMA. 2022;327(12):1158-1170. doi:10.1001/jama.2022.0992 35315886 PMC8941348

[37] van Kempen AAMW, Eskes PF, Nuytemans DHGM, ; HypoEXIT Study Group. Lower versus traditional treatment threshold for neonatal hypoglycemia. N Engl J Med. 2020;382(6):534-544. doi:10.1056/NEJMoa1905593 32023373

[38] Shah R, Harding J, Brown J, McKinlay C. Neonatal glycaemia and neurodevelopmental outcomes: a systematic review and meta-analysis. Neonatology. 2019;115(2):116-126. doi:10.1159/000492859 30408811

[39] Paudel N, Chakraborty A, Anstice N, ; CHYLD Study Group. Neonatal hypoglycaemia and visual development: a review. Neonatology. 2017;112(1):47-52. doi:10.1159/000456705 28253512 PMC5472486

