# Supplementary material for: Transitional Neonatal Hypoglycemia and Adverse Neurodevelopment in Midchildhood
Source: JAMA Netw Open. 2024 Mar 26;7(3):e243683. doi: 10.1001/jamanetworkopen.2024.3683 (PMC10966413; doi:10.1001/jamanetworkopen.2024.3683)
Supplement: Supplement 1. — eAppendix. Standard Operating Procedure for Neonatal Hypoglycemia at the University Children’s Hospital Düsseldorf, Germany, 2010-2015 eTable 1. Association of Neonatal Hypoglycemia and Neurodevelopmental Outcomes in Midchildhood (Unadjusted Data) eTable 2. Neurodevelopmental Outcomes After Singular or Recurrent Hypoglycemia ≤30 mg/dL [file jamanetwopen-e243683-s001.pdf]

Supplemental Online Content

Roeper M, Hoermann H, Körner LM, et al. Transitional neonatal hypoglycemia and adverse neurodevelopment in midchildhood. *JAMA Netw Open.* 2024;7(3):e243683. doi:10.1001/jamanetworkopen.2024.3683

eAppendix. Standard Operating Procedure for Neonatal Hypoglycemia at the University Children's Hospital Düsseldorf, Germany, 2010-2015

eTable 1. Association of Neonatal Hypoglycemia and Neurodevelopmental Outcomes in Midchildhood (Unadjusted Data)

eTable 2. Neurodevelopmental Outcomes After Singular or Recurrent Hypoglycemia ≤30 mg/dL

This supplemental material has been provided by the authors to give readers additional information about their work.

© 2024 Roeper M et al. *JAMA Network Open.*

eAppendix. Standard Operating Procedure for Neonatal Hypoglycemia at the University Children's Hospital Düsseldorf, Germany, 2010-2015

<u>Abbreviations:</u> GDM = Gestational Diabetes mellitus; DM = Diabetes mellitus; BG = Blood Glucose; LGA = Large for gestational age; SGA = Small for gestational age

<u>Screening:</u>
The following children should be screened for neonatal hypoglycemia:

1. **Maternal diabetes**: GDM; DM type 1; DM type 2; each controlled by medication or diet)

2. **LGA:** Macrosomic newborns >4200 g (birth weight >90th percentile)

3**. SGA**: Hypotrophic newborns <2600 g (birth weight <10th percentile)

4. **Late Preterm birth:** 35+0 – 36+6 weeks of gestation

5. **Every clinically conspicuous newborn** (tachycardia, shakiness, restlessness, sweating, etc.), regardless of the medical history

<u>Procedure:</u>
Babies at risk are transferred to the nursery within the first hour of life, receive a feed (within the first hour of life with pre-nutrition (= early feeding) and are given a blood glucose check after the first hour of life (blood glucose checks are not advisable before this!).

In addition, BG checks are carried out before the next 3 meals ("preprandial").

- **If BG is below 50 mg/dL:**
  - administer maltodextrin 10% 5 ml/kgKG and check BG again 30 minutes later.
  - Then continue the BG checks preprandial before the next 3 meals (= every 3 hours). Target: 3x BG >50mg/dL

- **If the BG is below 50 mg/dL again** (either at the check 30 minutes after hypoglycemia and after maltodextrin administration or in the course of the first 24 hours of life):
  - transfer to the pediatric clinic.

  - Start intravenous glucose administration. When administering intravenous glucose, boluses should be avoided. Start the maintenance requirement with 5 mg/kg/min, corresponding to a 10% glucose infusion at 3 ml/kg/h.

  - In parallel, start oral nutrition with SHORT feeding intervals = 8 meals, possibly even 10-12 meals in the first 24 hours.

Note: This protocol represents the institutional protocol for neonatal hypoglycemia at the University Children's Hospital Düsseldorf in the period 2010-2015 and does not represent any current consensus guideline.

© 2024 Roeper M et al. *JAMA Network Open.*

eTable 1. Association of neonatal hypoglycemia and neurodevelopmental outcomes in mid-childhood (unadjusted data)

Neurodevelopmental test	BG ≤30 mg/dL	BG >30 mg/dL		
	Mean (95 % CI)	*Mean (95 % CI)*	*Mean difference (95 % CI)*	*P value*
**WISC-V IQ** ***(index score)***[a]				
Total score	106.8 (103.4 - 110.2)	111.8 (108.6 - 115.0)	-4,9 (-9.5 to -.3)	.04
Verbal comprehension	108.8 (105.4 - 112.2)	113.5 (110.4 - 116.6)	-4.7 (-9.3 to -.1	.04
Visual spatial	103.3 (99.7 - 106.8)	107.0 (103.4 - 110.6)	-3.7 (-8.8 to 1.3)	.14
Fluid reasoning	105.9 (102.4 - 109.5)	108.2 (104.6 - 111.8)	-2.2 (-7.2 to 2.8)	.38
Working memory	105.7 (102.4 - 109.0)	107.5 (103.8 - 111.2)	-1.8 (-6.7 to 3.1)	.46
Processing speed	100.6 (97.0 - 104.2)	106.1 (102.9 - 109.2)	-5.5 (-10.3 to -.7)	.03
**MABC-2 motor function** ***(standard score)***[b]				
Total motor function	9.3 (8.6 - 10.0)	10.5 (9.9 - 11.0)	-1.2 (-2.1 to -.3)	.01
Fine motor function	9.3 (8.5 - 10.1)	10.7 (10.1 - 11.4)	-1.4 (-2.4 to -.4)	.007
Gross motor function	8.4 (7.7 - 9.1)	8.8 (8.1 - 9.5)	-.4 (-1.4 to .5)	.38
Balance	10.3 (9.6 - 11.0)	11.1 (10.5 - 11.7)	-.8 (-1.7 to .2)	.10
**DTVP Visual Perception** ***(index score)***[c]				
General visual perception	94.5 (91.1 - 97.9)	101.3 (97.7 - 104.9)	-6.8 (-11.7 to -1.9)	.009
Motor-reduced visual perception	96.6 (93.2 - 100.1)	99.6 (95.5 - 103.6)	-2.9 (-8.2 to 2.3)	.22
Visual-motor integration	93.2 (89.4 - 97.0)	103.2 (100.0 - 106.5)	-10.3 (-15.3 to -5.4)	<.001
**BRIEF** ***(parental rated T-scores)***[d] (n=137)				
Inhibit	50.3 (48.0 - 52.6)	48.9 (46.9 - 50.9)	1.3 (-1.7 to 4.4)	.62
Shift	47.7 (45.5 - 49.8)	49.0 (46.9 - 51.1)	-1.3 (-4.2 to 1.7)	.22
Emotional control	47.6 (45.4 - 49.8)	47.4 (45.5 - 49.3)	.2 (-2.7 to 3.1)	.88
Initiate	52.5 (49.6 - 55.4)	51.3 (49.1 - 53.6)	1.2 (-2.4 to 4.8)	.74
Working memory	51.7 (49.2 - 54.2)	48.3 (46.1 - 50.6)	3.3 (.02 to 6.7)	.03
Plan/organize	50.7 (48.5 - 52.8)	48.3 (46.1 - 50.5)	2.4 (-.6 to 5.4)	.06
Organization of materials	50.8 (48.5 - 53.2)	49.8 (47.2 - 52.5)	1.0 (-2.5 to 4.5)	.41
Monitor	49.7 (47.6 - 51.8)	49.5 (47.3 - 51.7)	.2 (-2.8 to 3.2)	.82

© 2024 Roeper M et al. *JAMA Network Open.*

eTable 1. Association of neonatal hypoglycemia and neurodevelopmental outcomes in mid-childhood (unadjusted data) continued

**Neurodevelopmental test**	**BG ≤30 mg/dL**	**BG >30 mg/dL**		
	Mean (95 % CI)	*Mean (95 % CI)*	*Mean difference (95 % CI)*	*P value*
**BRIEF** ***(parental rated T-scores)***[d] (n=137)				
Behavioral regulation index	48.6 (46.2 - 51.0)	48.2 (46.2 - 50.2)	.4 (-2.7 to 3.4)	.75
Metacognition index	51.2 (48.9 - 53.5)	49.3 (47.0 - 51.6)	1.9 (-1.3 to 5.1)	.18
Global executive composite	50.3 (48.0 - 52.5)	48.6 (46.5 - 50.8)	1.6 (-1.5 to 4.7)	.33
**CBCL** ***(parental rated T-scores)***[e] (n=136)				
Syndrome scales				
Anxious/depressed	56.8 (55.0 - 58.6)	56.5 (54.9 - 58.1)	.3 (-2.1 to 2.6)	.82
Depressed	55.2 (53.6 - 56.9)	53.1 (52.0 - 54.2)	2.1 (.1 to 4.1)	.15
Somatic complaints	55.2 (53.5 - 56.9)	55.5 (53.9 - 57.1)	-.3 (-2.6 to 1.9)	.61
Social problems	55.9 (54.1 - 57.7)	54.9 (53.5 - 56.3)	1.0 (-1.2 to 3.3)	.43
Thought problems	57.6 (56.0 - 59.3)	56.4 (54.8 - 58.0)	1.2 (-1.1 to 3.5)	.22
Attention problems	58.1 (55.9 - 60.2)	55.0 (53.0 - 56.9)	3.1 (.2 to 6.0)	.006
Rule-breaking behavior	54.7 (53.3 - 56.1)	55.3 (53.9 - 56.6)	-.6 (-2.5 to 1.3)	.47
Aggressive behavior	55.1 (53.2 - 57.1)	54.2 (52.7 - 55.7)	.9 (-1.5 to 3.3)	.70
Internalizing	54.1 (51.6 - 56.5)	53.0 (50.9 - 55.0)	.8 (-2.3 to 3.9)	.83
Externalizing	51.1 (48.5 - 53.7)	50.6 (48.3 - 53.0)	.2 (-3.2 to 3.6)	.90
Total problems	53.9 (51.3 - 56.5)	51.7 (49.5 - 53.9)	1.9 (-1.5 to 5.3)	.27
DSM-oriented scales				
Affective problems	55.8 (53.9 - 57.6)	53.8 (52.6 - 55.0)	2.0 (-.2 to 4.1)	.46
Anxiety problems	57.1 (55.5- 58.8)	57.1 (55.4 - 58.8)	.1 (-2.3 to 2.4)	.97
Somatic problems	54.8 (53.1 - 56.6)	54.7 (53.0 - 56.3)	.1 (-2.3 to 2.5)	.96
ADHD	58.1 (55.9 - 60.2)	54.9 (53.2 - 56.7)	3.1 (.3 to 5.9)	.02
Oppositional defiant problems	54.8 (53.1 - 56.5)	54.6 (53.1 - 56.2)	.2 (-2.1 to 2.5)	.95
Conduct problems	55.2 (53.5 - 56.8)	54.9 (53.5 - 56.4)	.3 (-1.9 to 2.4)	.99

© 2024 Roeper M et al. *JAMA Network Open.*

eTable 1. Association of neonatal hypoglycemia and neurodevelopmental outcomes in mid-childhood (unadjusted data) footnotes

[a] Wechsler's Intelligence Scale for Children - fifth edition (WISC-V): standardized mean (SD) 100 (15). Higher scores indicate higher IQ.
[b] Movement Assessment Battery for Children - second edition (MABC-2): standardized mean (SD) 10 (3). Higher scores indicate higher function.
[c] Developmental Test of Visual Perception - third edition/adolescent and adult (DTVP-3, DTVP-A): standardized mean (SD) 100 (15). Higher scores indicate higher function.
[d] Behavior Rating Inventory of Executive Function (BRIEF): standardized t-score mean (SD) 50 (10). Higher scores indicate greater problems.
[e] Child Behavior Checklist (CBCL): standardized t-score mean (SD) 50 (10). Higher scores indicate greater problems.
Abbreviations: ADHD = Attention Deficit Hyperactivity Disorder. BG = Blood Glucose. 95% CI = 95% Confidence Interval. DSM = Diagnostic and Statistical Manual of Mental Disorders. IQ = Intelligence Quotient. N = number (only reported in case of missing data). % = Percent. SD = Standard Deviation.
SI conversion factor: To convert blood glucose concentration to millimoles per liter, multiply values by 0.0555.

© 2024 Roeper M et al. *JAMA Network Open.*

eTable 2. Neurodevelopmental outcomes after singular or recurrent hypoglycemia ≤30 mg/dL

**Neurodevelopmental test**	**Recurrent BG ≤30 mg/dL (n = 21)**	**Singular BG ≤30 mg/dL (n = 49)**		
	Mean (95 % CI)	*Mean (95 % CI)*	*Mean difference (95 % CI)*	*P value*
**WISC-V IQ *(index score)*[a]**				
Total score	106.2 (99.4 - 113.0)	107.1 (103.1 - 111.2)	.9 (-6.5 to 8.4)	.80
Verbal comprehension	110.3 (103.1 - 117.4)	108.2 (104.2 - 112.1)	-2.1 (-9.6 to 5.3)	.57
Visual spatial	100.9 (93.0 - 108.9)	104.3 (100.3 - 108.2)	3.4 (-4.4 to 11.1)	.39
Fluid reasoning	105.6 (98.2 - 113.0)	106.1 (101.9 - 110.2)	.5 (-7.4 to 8.3)	.91
Working memory	105.1 (98.0 - 112.3)	105.9 (102.1 - 109.7)	.8 (-6.5 to 8.0)	.84
Processing speed	97.5 (90.6 - 104.3)	101.9 (97.5 - 106.4)	4.5 (-3.5 to 12.4)	.27
**MABC-2 motor function *(standard score)*[b]**				
Total motor function	8.8 (7.6 - 10.0)	9.5 (8.6 - 10.5)	.7 (-.8 to 2.3)	.36
Fine motor function	8.7 (7.4 - 10.0)	9.6 (8.5 - 10.6)	.9 (-.8 to 2.7)	.31
Gross motor function	8.3 (7.0 - 9.6)	8.4 (7.5 - 9.3)	.1 (-1.5 to 1.7)	.90
Balance	10.4 (8.9 - 12.0)	10.2 (9.4 - 11.1)	-.2 (-1.8 to 1.4)	.82
***DTVP Visual Perception (index score)*[c]**				
General visual perception	91.0 (85.3 - 96.7)	96.1 (91.8 - 100.3)	5.1 (-2.3 to 12.4)	.17
Motor-reduced visual perception	94.0 (87.1 - 100.0)	97.8 (93.8 - 101.8)	3.8 (-3.6 to 11.2)	.31
Visual-motor integration	90.1 (84.9 - 95.3)	94.6 (89.6 - 99.6)	4.0 (-4.2 to 12.2)	.33

[a] Wechsler's Intelligence Scale for Children - fifth edition (WISC-V): standardized mean (SD) 100 (15). Higher scores indicate higher IQ.
[b] Movement Assessment Battery for Children - second edition (MABC-2): standardized mean (SD) 10 (3). Higher scores indicate higher function.
[c] Developmental Test of Visual Perception - third edition/adolescent and adult (DTVP-3, DTVP-A): standardized mean (SD) 100 (15). Higher scores indicate higher function.
Abbreviations: BG = Blood Glucose. IQ = Intelligence Quotient. 95% CI = 95% Confidence Interval. SD = Standard Deviation.
Recurrent hypoglycemia ≤30 mg/dL was defined as two or more episode below the cutoff. Exploratory unadjusted data are reported.
SI conversion factor: To convert blood glucose concentration to millimoles per liter, multiply values by 0.0555.

© 2024 Roeper M et al. *JAMA Network Open.*
