# Supplementary material for: Transitional Neonatal Hypoglycemia and Adverse Neurodevelopment in Midchildhood
Source: JAMA Netw Open. 2024 Mar 26;7(3):e243683. doi: 10.1001/jamanetworkopen.2024.3683 (PMC10966413; doi:10.1001/jamanetworkopen.2024.3683)
Supplement: Supplement 2. — Data Sharing Statement [file jamanetwopen-e243683-s002.pdf]

Data Sharing Statement

Roeper. Transitional Neonatal Hypoglycemia and Adverse Neurodevelopment in Midchildhood. *JAMA Netw Open*. Published March 26, 2024. doi:10.1001/jamanetworkopen.2024.3683

Data
Data available: Yes
Data types: Deidentified participant data
How to access data: Pseudonymized raw data that underlie the results reported in this article may be made available upon reasonable request to the corresponding author via email: marcia.roeper@med.uni-duesseldorf.de
When available: With publication

Supporting Documents
Document types: None

Additional Information
Who can access the data: Researchers whose proposed use of the data has been approved.
Types of analyses: For a specified purpose.
Mechanisms of data availability: After approval of a proposal.
